# The clinical application of V-Y advanced flap pedicled with freestyle perforator flap for repairing small range defects in the anterior knee region

**DOI:** 10.3389/fsurg.2024.1364340

**Published:** 2024-05-13

**Authors:** Da Qian, Lijie Jin, Guoxin Huang, Ping Dai, Dong Li, Hui Lu, Ming Xu, Ke Wang, Xian Zhong, Xiaochen Xu, Jianchao Zhang, Bin Yu

**Affiliations:** ^1^ Department of Burn and Plastic Surgery-Hand Surgery, Changshu Hospital Affiliated to Soochow University, Changshu No.1 People’s Hospital, Changshu, China; ^2^ Department of Nursing, Changshu Hospital Affiliated to Soochow University, Changshu No.1 People’s Hospital, Changshu, China; ^3^ Department of Evidence-Based Medicine Center, Xiangyang No.1 People’s Hospital, Hubei University of Medicine, Xiangyang, China

**Keywords:** V-Y advanced flap, anterior region of knee, soft tissue injury, freestyle perforator flap, wound healing

## Abstract

**Introduction:**

This study aims to investigate the clinical efficacy of V-Y advanced flap pedicled with freestyle perforator flap for repairing small range defects in the anterior knee region.

**Methods:**

8 patients with skin and soft tissue defect/necrosis in the anterior knee area admitted to the Changshu No.1 People's Hospital from January 2021 to January 2022 were selected, with a defect range of 4 cm × 3 cm–9 cm × 6 cm, designed a V-Y advanced flap pedicled with freestyle perforator flap to repair the wound in the anterior knee area. Adjust the size and position of the flap according to the number and position of perforating branches found during the surgery, with a cutting area of 6 cm × 5 cm–14 cm × 10 cm and the supply area was directly pulled and sutured.

**Results:**

4 patients were repaired by flaps pedicled with 2 perforating branches, 2 patients were repaired by flaps pedicled with 1 perforating branch and 2 patients were repaired by flaps pedicled with 3 perforating branches. 4 patients were repaired by flaps pedicled with 2 perforating branches, 2 patients were repaired by flaps pedicled with 1 perforating branch and 2 patients were repaired by flaps pedicled with 3 perforating branches. All flaps survived and following up for 6-15 months, the blood supply, appearance, and color of the flap were satisfactory, and the functions of knee joint flexion and extension were well preserved.

**Discussion:**

The V-Y advancement flap pedicled with freestyle perforator flap has the advantages of reliable blood supply, simple surgical operation, texture and thickness similar to the skin of the anterior knee area, and direct suture of the donor area. It is a perforator flap with good repair effect for small scale defects in the anterior knee area.

## 1 Introduction

Anterior knee injuries commonly occur in the clinical setting and often result from trauma or burns (1, 2). Due to the sparse soft tissue in the Anterior knee region, skin necrosis is a frequent issue (3). This area plays a crucial role in joint mobility; thus, maintaining aesthetic appearance and knee joint mobility presents a significant challenge to plastic surgeons (4). Direct suturing or grafting is typically not the preferred approach to treat these injuries; instead, flap repair is often used. This type of injury is usually repaired with a skin flap, including perforator, fascial, muscle, and musculocutaneous flaps (5). However, this method frequently leaves secondary wounds that require grafting (6, 7).

In the field of plastic and reconstructive surgery, a variety of techniques are commonly employed to manage soft tissue injuries and defects. Among these, the use of flap repair, including perforator flaps, has been well-documented (8). Despite their efficacy, these methods can leave secondary wounds that necessitate further grafting. Consequently, there is a constant need for surgical techniques that minimize such secondary complications while ensuring effective wound repair and patient recovery (8). Various experts have conducted extensive discussions and research on flap repairing in this area (1, 7, 9). However, each type of flap has its advantages and disadvantages. Local flaps offer limited soft tissue coverage, making it difficult to achieve optimal repair for larger Anterior knee area defects (10). Free flaps can provide superior tissue coverage but cause significant donor site morbidity and are often bulky, which is unfavorable for subsequent knee joint function recovery (10).

Our aim is to identify a surgical method that can be promoted in primary care hospitals to treat small Anterior knee area defects. In this study, we utilized freestyle-pattern V-Y advancement flaps to repair small Anterior knee area defects. Moreover, we discuss the preparation techniques, therapeutic effects, and complications of freestyle-pattern V-Y advancement flaps in this article.

## 2 Clinical data and surgical technique

### 2.1 Clinical data

The study included two male and six female patients, with ages ranging from 21 to 65 years (average 46 years). The time from injury to hospital admission ranged from 2 h to 7 days. Two cases of car accident injuries, four cases of crushing injuries, and two cases of avulsion injury. The wound area ranged from 4 cm × 3 cm to 9 cm × 6 cm. There were no undermining injuries around the wound, and three cases had patellar or tendon exposure. Imaging showed no significant fractures or osteomyelitis.

### 2.2 Anatomical basis of flaps and surgical techniques

The knee joint region has an abundant blood supply to the soft tissues of the skin, primarily from the muscular branches of the femoral artery, the genicular artery, and the superior and inferior medial and lateral genicular arteries. These arteries form an extensive anastomotic network, which serves as the vascular foundation for the survival of various pedicled flaps in the knee joint area (11).

The genicular artery usually originates from the femoral artery or occasionally from the popliteal artery and frequently gives off hidden and articular branches. The genicular artery has constant perforators that are closely connected to the surrounding vascular network. Each artery's contribution to skin nutrition has been quantified, providing the anatomical basis for the design and use of perforator flaps in the knee area.

In this study, flap repair surgery was performed 3 h–7 days after admission. The “V”-shaped flap was designed in a relatively relaxed area around the pre-knee wound, with the edge of the wound as the base. The length of the base was slightly longer than the width of the wound by about 1 cm, and the length of the flap was usually 2–3 times the width of the wound.

The procedure was performed under general anesthesia, with the patient in a supine position and the affected limb extended. A pneumatic tourniquet was applied at the root of the thigh. Thorough debridement of the wound was performed. The skin, subcutaneous tissue, and deep fascia on one side of the flap were first cut along the preoperative design line, and the flap was lifted from the deep fascia to the opposite side, carefully looking for perforators and protecting them properly.

The position and size of the flap were adjusted appropriately according to the number (as many as possible to ensure blood supply to the flap), position, and thickness of the perforators found, and large perforators located in the center of the flap were retained to ensure blood supply to the flap and increase the advancement distance of the flap.

The other side of the flap and the edge of the wound was cut in sequence, taking care to protect the nerve branches entering the flap. In this group of patients, the flap area was 6 cm × 5 cm–14 cm × 10 cm. The flap was freed under the deep fascia until only the perforators and cutaneous nerves supplying the flap were connected to the flap.

The tourniquet was released to observe the blood supply of the flap and to achieve complete hemostasis simultaneously. The “V”-shaped flap was advanced to cover the wound, and the wound edge was sutured in layers. The donor area was directly sutured, and the wound appeared “Y”-shaped after suturing. A drain was placed under the flap and connected to a negative pressure ball, routinely drained for 3–5 days, and removed as appropriate.

### 2.3 Postoperative management

Postoperatively, the knee joint was routinely fixed with plaster until the suture was removed. Symptomatic treatments, including antibiotics for infection control, circulation improvement measures, anticoagulants, and muscle relaxants, were routinely administered. Observations were made on the time of capillary filling, color, and surface temperature of the skin flap to evaluate arterial perfusion and venous return of the flap. The sutures were removed after 2–3 weeks postoperatively.

## 3 Typical case

The patient, a 37-year-old male, was admitted to the hospital 4 h after suffering from pain and bleeding due to an avulsion injury to his left knee caused by a fall. The skin defect area in the anterior region of the left knee was approximately 4 cm × 6 cm. The marginal abrasions were severe, and the wound was significantly contaminated. The ligament was exposed at the base, and no significant fracture signs were observed on the radiographs. Emergency debridement and VSD treatment were performed. The flap repair surgery was performed one week later.

In the preoperative design, a V-Y advancement flap with freestyle perforator as the pedicle was planned. After cutting the flap along the design line (the area was 12 cm × 7 cm), two perforators were found connected to the flap. The flap was advanced to repair the anterior knee wound, and the donor area was directly closed by suturing. Postoperative management included routine antibiotic administration, muscle relaxation, and anticoagulant treatment. After 72 h postoperatively, the flap survived. The sutures were removed two weeks postoperatively, and both the donor and recipient areas healed well.

**Figure 1:**
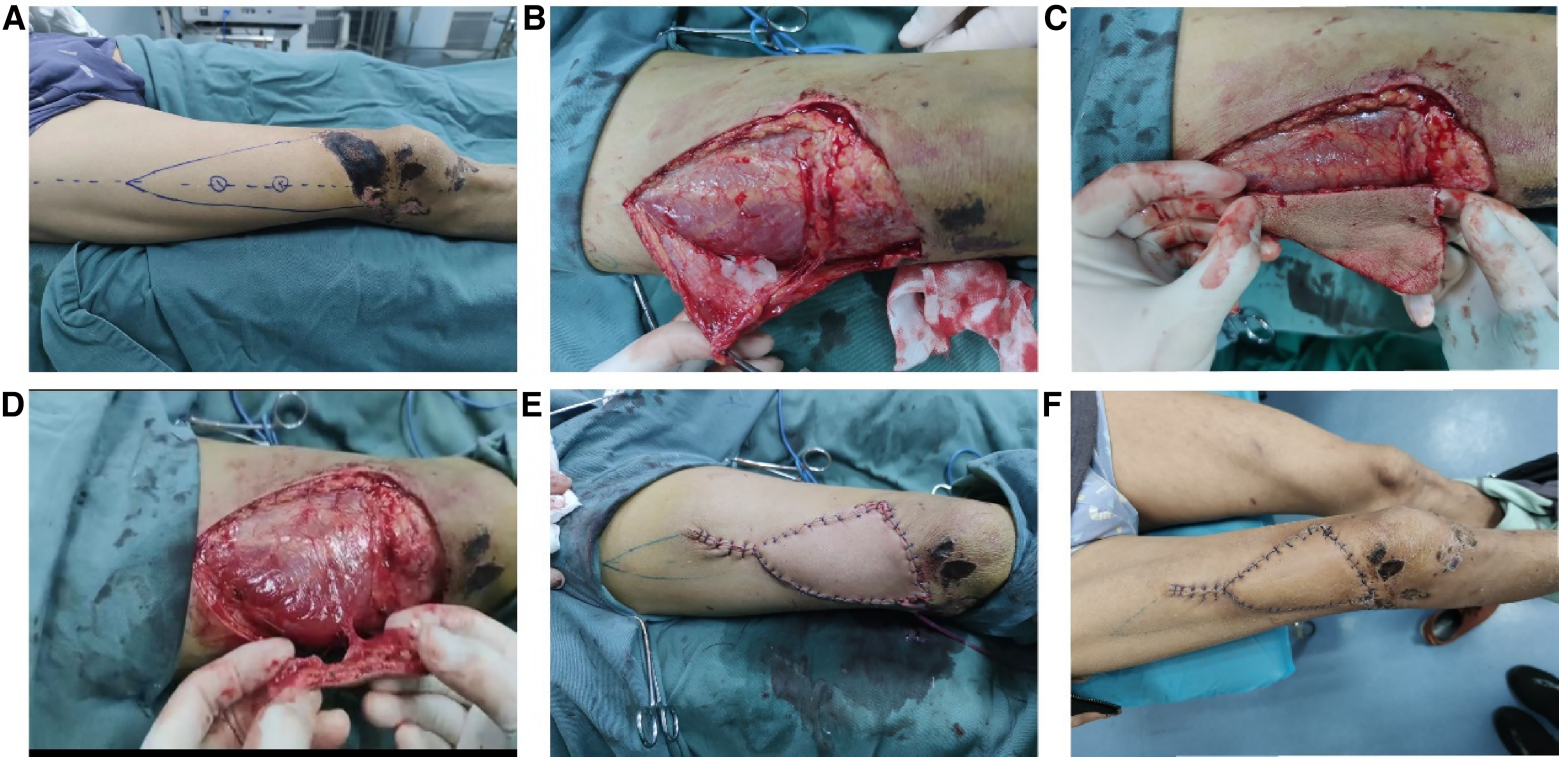
A typical case of repairing a small area of defect/necrosis in the anterior knee region using a V-Y flap with arbitrary perforator pedicle. The flap design (**A**), the surgical procedure of flap harvesting (**B**,**C**,**D**), the flap covering the defect area (**E**), and the condition of the flap 14 days postoperatively (**F**).

**Figure 2:**
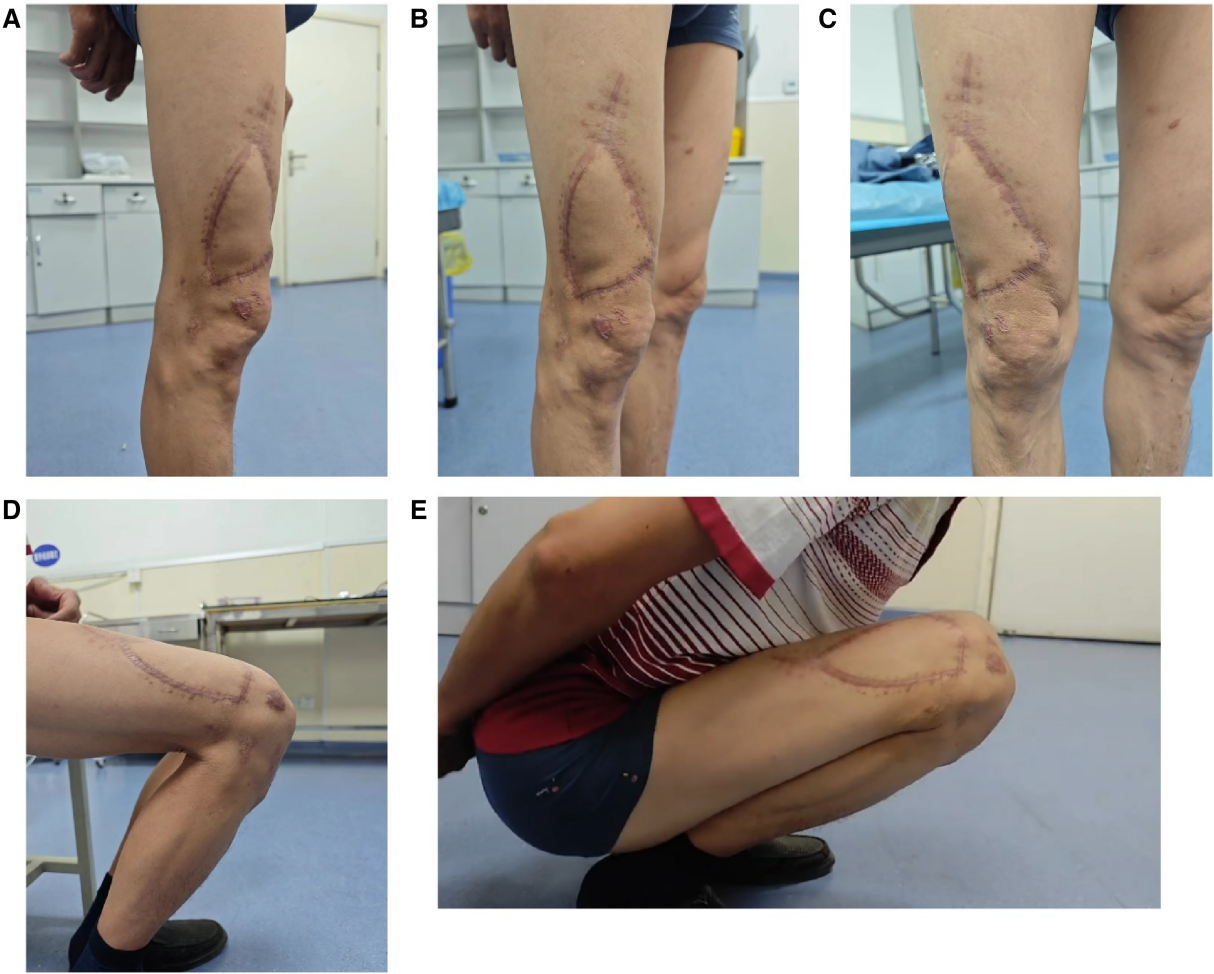
Follow-up images of a representative case at 8 months postoperative. Side view (**A**), 45° oblique view (**B**), frontal view (**C**), knee joint flexed at 90° (**D**), knee joint flexed at 180° (**E**).

At the one-month follow-up, the flap showed good color and texture, with a pleasing appearance, but slightly weak sensation; the flexion and extension functions of the knee joint were good (see Figure 1). An eight-month follow-up is shown in Figure 2.

## 4 Results

In this group, four patients had flaps based on two perforators, two patients had flaps based on one perforator, and two patients had flaps based on three perforators. All flaps in this group survived at 72 h postoperatively, and both the donor and recipient site wounds healed at two weeks postoperatively. During the 1–12-month follow-up period, with an average follow-up of 8 months, the flaps showed no signs of congestion, had improved appearance, and slightly diminished sensation. The knee joint demonstrated good flexion and extension function. The patients’ conditions are outlined in Table 1.

**Table 1:** The status of all of the 8 enrolled patients.

Patient number	Gender	Age	Defect area (cm)	Intraoperative blood loss (ml)	Postoperative drainage tube (day)	Flap outcome
1	Female	21	4 × 8	37	3	Survived
2	Female	42	3 × 6	40	4	Survived
3	Male	37	4 × 6	37	4	Survived
4	Female	45	4 × 9	43	3	Survived
5	Female	38	3 × 7	48	5	Survived
6	Female	58	3 × 6	49	3	Survived
7	Male	62	4 × 7	50	4	Survived
8	Female	65	4 × 8	36	5	Survived

## 5 Discussion

The knee joint, being the largest, most complex, and highest weight-bearing joint in the human body, is prone to soft tissue defects around it due to factors such as trauma and infection (12). These injuries often involve exposed bones, joints, or ligaments, presenting significant challenges to clinicians. Several methods have been devised to repair soft tissue defects in the knee joint, each with their strengths and limitations (13–16). As the standards for wound repair escalate, the flap is required to not only repair the wound and minimize destruction to the donor area but also to retain functionality and meet high aesthetic demands (1).

Through literature review and understanding of the blood supply anatomy around the knee joint, we designed a V-Y advancement flap with arbitrary perforators as the pedicle to repair a small wound in the anterior knee area. This design leverages the advantages of traditional V-Y advancement flaps, offering small postoperative scar and direct closure of the donor area. The perforators, often unnamed, are arbitrary, and preoperative detection with a Doppler blood flow detector is not required (17). This flap does not cause damage to the main blood vessels and muscles, resulting in minimal destruction to the donor area. If necessary, moderately freeing the perforators towards the deep layer can increase the advancement distance of the flap, with the furthest advancement in our cases being 6 cm.

This flap is relatively simple to operate for surgeons, not requiring microsurgical techniques to anastomose vessels or nerves, making it suitable for clinical application in grassroots hospitals in China. The flap donor area can be directly sutured, avoiding the disadvantages of graft repair such as pigmentation, contraction, and scarring in the graft area. By using an advancement method to repair the wound, the flap avoids torsion and compression at the pedicle, ensuring the blood supply to the flap to the greatest extent.

The limitation of this flap is that it is only suitable for repairing smaller anterior knee wounds (advancement distance less or equal to 6 cm) and is not applicable for defects with deep cavity-like wounds. We consider wounds with an advancement distance greater than 6 cm or smaller than 2 cm unsuitable for this method. For wounds with an advancement distance smaller than 2 cm, the wound edge can be moderately freed and directly sutured, and postoperative influence on the knee joint activity is not significant. For wounds with an advancement distance larger than 6 cm, advancement may not fully cover the wound. Another limitation is the number and individual differences of perforators. Due to intraoperative manipulation and variation, the flap design may need to be adjusted according to the position of the perforators, which introduces some uncertainty.

During the treatment process, we must pay attention to ensure that there is no lurking avulsion injury around the wound preoperatively. One side of the flap is first incised, and the flap is lifted under the deep fascia to find the perforators and adjust the flap according to the quantity and thickness of the perforators. The quantity and size of the perforators vary from person to person, and special consideration is needed during the operation. During the operation, try to preserve as many perforators as possible to increase the blood supply to the flap, but too many perforators can affect the advancement distance of the flap. If you free the perforators towards the deep layer to increase the advancement distance, it will increase local damage and the operation time. According to our center's recommendation, a maximum of 3 perforators should be preserved. If a thicker perforator is observed intraoperatively, considering the advancement distance of the flap, other perforators can be ligated, and only 1 or 2 perforators are preserved. The subcutaneous tissue at the edge of the flap is sutured and fixed to the deep fascia to prevent separation that may affect the flap's blood supply. Finally, the flap is sutured in layers to reduce skin tension and minimize postoperative scarring.

## 6 Conclusion

In conclusion, our findings suggest that the V-Y advancement flap with arbitrary perforators as the pedicle exhibits substantial reliability in blood supply, simplicity in surgical operation, and mimicry of the texture and thickness of the anterior knee skin. In addition, the donor area can be directly sutured, which further enhances its applicability. This flap offers an effective approach to repairing smaller defects in the anterior knee area. Given its benefits and practicality, it merits wider adoption and promotion in clinical practice, particularly within grassroots hospitals. The technique represents an important addition to our armamentarium in reconstructive surgery, contributing to improved patient outcomes, and further research and clinical application are warranted.

## Data Availability

The original contributions presented in the study are included in the article/Supplementary Material, further inquiries can be directed to the corresponding authors.
